# The targeted SMAC mimetic SW IV-134 augments platinum-based chemotherapy in pre-clinical models of ovarian cancer

**DOI:** 10.1186/s12885-022-09367-w

**Published:** 2022-03-12

**Authors:** Pratibha S. Binder, Yassar M. Hashim, James Cripe, Tommy Buchanan, Abigail Zamorano, Suwanna Vangveravong, David G. Mutch, William G. Hawkins, Matthew A. Powell, Dirk Spitzer

**Affiliations:** 1 Division of Gynecologic Oncology, Department of Obstetrics and Gynecology, Washington University School of Medicine, 660 S. Euclid Ave, Box 8109, Saint Louis, MO 63110 USA; 2 Present Address: Rebecca and John Moores Cancer Center, 3855 Health Science Drive, La Jolla, CA USA; 3 Department of Surgery, Washington University School of Medicine, St. Louis, MO USA; 4 Present Address: Cedars Sinai Medical Center, 8635 W. 3rd Street, Los Angeles, CA USA; 5 Alvin J. Siteman Cancer Center, St. Louis, MO USA

**Keywords:** Sigma-2 receptors, Sigma-2/SMAC drug conjugate, Cisplatin, Combination therapy, Ovarian cancer

## Abstract

**Background:**

Ovarian cancer is initially responsive to frontline chemotherapy. Unfortunately, it often recurs and becomes resistant to available therapies and the survival rate for advanced and recurrent ovarian cancer is unacceptably low. We thus hypothesized that it would be possible to achieve more durable treatment responses by combining cisplatin chemotherapy with SW IV-134, a cancer-targeted peptide mimetic and inducer of cell death. SW IV-134 is a recently developed small molecule conjugate linking a sigma-2 ligand with a peptide analog (mimetic) of the intrinsic death pathway activator SMAC (second-mitochondria activator of caspases). The sigma-2 receptor is overexpressed in ovarian cancer and the sigma-2 ligand portion of the conjugate facilitates cancer selectivity. The effector portion of the conjugate is expected to synergize with cisplatin chemotherapy and the cancer selectivity is expected to reduce putative off-target toxicities.

**Methods:**

Ovarian cancer cell lines were treated with cisplatin alone, SW IV-134 alone and a combination of the two drugs. Treatment efficacy was determined using luminescent cell viability assays. Caspase-3/7, − 8 and − 9 activities were measured as complementary indicators of death pathway activation. Syngeneic mouse models and patient-derived xenograft (PDX) models of human ovarian cancer were studied for response to SW IV-134 and cisplatin monotherapy as well as combination therapy. Efficacy of the therapy was measured by tumor growth rate and survival as the primary readouts. Potential drug related toxicities were assessed at necropsy.

**Results:**

The combination treatment was consistently superior in multiple cell lines when compared to the single agents in vitro. The expected mechanism of tumor cell death, such as caspase activation, was confirmed using luminescent and flow cytometry-based assay systems. Combination therapy proved to be superior in both syngeneic and PDX-based murine models of ovarian cancer. Most notably, combination therapy resulted in a complete resolution of established tumors in all study animals in a patient-derived xenograft model of ovarian cancer.

**Conclusions:**

The addition of SW IV-134 in combination with cisplatin chemotherapy represents a promising treatment option that warrants further pre-clinical development and evaluation as a therapy for women with advanced ovarian cancer.

**Supplementary Information:**

The online version contains supplementary material available at 10.1186/s12885-022-09367-w.

## Background

The majority of patients diagnosed with ovarian, fallopian or primary peritoneal cancer, commonly referred to as Mullerian cancer, present with advanced stage disease [1]. Primary treatment includes a combination of cytoreductive surgery and systemic chemotherapy. Upfront surgery followed by chemotherapy or interval surgery after several cycles of chemotherapy have been employed as standard therapeutic options. Chemotherapy followed by surgery increases the likelihood of complete resection with no gross residual cancer behind at the surgical sites with acceptable morbidity [2–4]. The recommended first line chemotherapies include platinum- and taxane-based regimens, both via intravenous (IV) and intraperitoneal (IP) administration routes [5–7]. Recently, an anti-angiogenic drug, bevacizumab, was approved in combination with chemotherapy as a maintenance regimen for patients with stage III or IV epithelial Mullerian cancer after initial surgical resection. This combination led to a modest improvement in progression-free survival, but overall survival benefit was only seen in patients with high-risk disease [8, 9]. Also, therapies targeting the DNA replication machinery of the cells with Poly (ADP-ribose) polymerase inhibitors (PARP-inh) have been approved as maintenance regimen in patients with and without homologous recombination repair deficiency (HRD) and has significantly improved survival in patients with HRD [10–12].

Most ovarian cancer patients tolerate initial chemotherapy well. However, 10–58% of patients do not complete the initial six-cycle regimen due to severe toxicities, including thrombocytopenia, neutropenia, gastrointestinal symptoms, neuropathy and other drug-related reactions [5–7]. These toxicities may result in dose delays, dose reductions, changes in chemotherapy regimen, or the addition of medications for bone marrow support. The majority of patients will achieve a complete clinical response to primary treatment; unfortunately, 70% will recur within 3 years, and over 85% will recur within 5 years after diagnosis [13–15]. If recurrence starts more than 6 months after completion of primary therapy, the recommended follow-up treatment is platinum-based combination therapy. While second-line treatment is available, it is limited due to increased toxicity and decreased efficacy.

Apoptosis represents an important mechanism of cancer cell death but is often blocked during disease initiation and progression [16]. More specifically, the X-linked inhibitor of apoptosis proteins (XIAP), is a potent negative regulator of the apoptotic pathways involving caspases-3, − 7 and − 9 blockade and thus promotes cancer cell survival via overexpression [17–19]. As such, down-modulation of XIAP activity has been studied as a mechanism to increase apoptosis and to overcome continued cell proliferation in vitro and in preclinical mouse models of ovarian cancer [20–22]. Second mitochondria-derived activator of caspases (SMAC) is an endogenous negative regulator of inhibitors of apoptosis proteins, including XIAP and cellular IAP (cIAP) and, in doing so, restores caspase activity and cancer cell death [23]. These findings have initiated the development of synthetic small molecule mimics of endogenous SMAC protein, which have been studied in a wide variety of human malignancies, including ovarian cancer, either as single agents or in combination with platinum-based therapies as a means to further improve patient outcomes [24–29].

In an attempt to further improve the therapeutic index of cancer drugs and to minimize off-site toxicities, our laboratory has developed a drug delivery concept that is based on the chemical conjugation of small molecule compounds, such as the SMAC mimetic SW IV-52, to ligands, e.g. SW43 to the sigma-2 receptor - highly upregulated in a number of solid tumors, including ovarian cancer [30]. This conjugation process resulted in a novel chemical entity, SW IV-134, that combines an improved internalization efficacy into the cancer cells with superior cytotoxicity, mediated via the distinct structural domains of the dual-functional drug conjugate and represents a pure enantiomer, reflecting the exact structural conformation as the SMAC mimetic SW IV-52 [31] in contrast to a racemic mix (SW III-123) that has been reported earlier [32]. As a result, SW IV-134 turned out to be ~ 2-fold more active than SW III-123 in SKOV-3 ovarian cancer cells in vitro (D. Spitzer, personal communication). Recently, we have shown that SW IV-134 induced much stronger cytotoxicity than its individual components administered as equimolar mixes, decreased the tumor burden and improved animal survival in a mouse xenograft model of ovarian cancer [31]. Since one of the limitations of platinum-based chemotherapy is significant systemic toxicity and cancer cell resistance, we sought to demonstrate that the targeted SMAC mimetic SW IV-134 in combination with low-dose cisplatin chemotherapy would provide efficient treatment benefits while systemic toxicities are reduced to a minimum.

## Methods

### Compounds

The synthesis of SW IV-134 was performed in our laboratory and has been previously described [31, 32]. Cisplatin was purchased from the pharmacy at Washington University School of Medicine.

### Cell lines

OVCAR-3 cells were purchased from American Type Culture Collection (ATCC, Manassas, VA) and cultured under ATCC-recommended conditions. SKOV-3 cells obtained from Dr. Robert Mach (Washington University School of Medicine, St. Louis, MO) were maintained in McCoy′s 5a medium containing 2 mM Glutamine and 10% Fetal Bovine Serum (FBS). ID8 mouse ovarian surface epithelial cells (MOSEC) obtained from Dr. Kathy Roby (Kansas University Medical Center, Kansas City, KS) were maintained in Dulbecco’s Modified Eagle’s medium (DMEM, Gibco-Life Technologies) containing 4% FBS. ID8 cells were labeled with eYFP/luciferase reporter fusion protein by retroviral infection to generate ID8-Luey cells. Protein expression was confirmed in 75% of the cells by flow cytometry and in vitro luciferin conversion. Antibiotics, penicillin (100 μg/mL) and streptomycin (100 μg/mL) were added to the media. Cells were maintained in a humidified incubator at 37 °C with 5% CO_2._ All cell lines were confirmed to be *mycoplasma*-negative prior to initiation of experiments.

### Mice

C57BL/6 mice, NSG and NOD.CB17-PRKDSCID mice were obtained from Jackson Laboratory at age 6–8 weeks. Injection of tumor cells or transplant of tumor tissues was performed no sooner than 1 week after the mice were received. All animal experimentation was performed in accordance with the Washington University Division of Comparative Medicine guidelines for care and use of laboratory animals. The protocol was approved by the Animal Studies Committee of Washington University (protocol 20,130,073). End points for euthanasia included excessive lethargy, decreased motility, tumor ulceration or cross-sectional tumor diameter greater than 2 cm.

### Evaluation of cytotoxicity in vitro

SKOV-3 cells were plated at a density of 1 × 10^4^/well, OVCAR-3 at a density of 1.5 × 10^4^/well and ID8 at a density of 3 × 10^3^/well in 96-well plates for 24 h prior to treatment. Cisplatin was dissolved in PBS to achieve a concentration of 5 μg/mL. SW IV-134 was dissolved in dimethyl sulfoxide (DMSO) and diluted in culture medium to achieve a final concentration of 0.25 μM for SKOV-3 cells, 4 μM for OVCAR-3 cells and 2 μM for ID8 cells (DMSO concentration was kept below 1% to have no impact on experimental results). Cells were treated with cisplatin, SW IV-134, and a combination of the two drugs for 72 h (SKOV-3 and OVCAR-3) and for 36 h (ID8), respectively. Cell viability was determined using CellTiter-Glo Luminescent Viability Assay (Promega, Madison, WI). Luminescence signal was measured using a multi-mode microplate reader (Bio-Tek, Winooski, VT). All assays were performed in triplicates.

### In vitro caspase activation assays

ID8 cells were plated at a density of 3 × 10^3^ in 96-well plates for 24 h prior to treatment. The following day, the cells were treated with 5 μg/mL cisplatin, 1 μM SW IV-134, a combination of the two drugs, and DMSO-containing media as a control for 48 h. The contents of the plate were mixed using an orbital shaker for 30 s and incubated at room temperature for 90 min. Caspase-3/7, − 8 and − 9 activities were measured in the plates using Caspase-Glo Assay Systems (Promega, Madison, WI) according to the manufacturer’s instructions. This assay is based on luminogenic caspase substrates which are cleaved by activated caspases resulting in generation of a luminescence signal. Luminescence signals were measured using a multi-mode microplate reader (Bio-Tek, Winooski, VT).

### In vivo assessment of tumor growth, survival, and toxicity in C57BL/6 mouse model

C57BL/6 mice were injected in the right flank with 200 μL single cell suspension of 1 × 10^7^ ID8-Luey cells in DMEM medium. Treatment started after ~ 4 weeks when tumors were established to be growing and reached 6–7 mm in diameter. Mice were randomized into four groups with 10 mice per group (*n* = 10). Treatment included intraperitoneal injection of 100 μL of vehicle daily (25% cremophor-EL in water), SW IV-134 (500 nmoles [17 mg/kg]) daily, cisplatin (2 mg/kg) every 3 days or combination of SW IV-134 (500 nmoles [17 mg/kg]) daily and cisplatin (2 mg/kg) every 3 days for a total of 21 days. On the days mice received both SW IV-134 and cisplatin, and as a preventive measure, the injections were given at least 2 h apart in case of potential drug incompatibilities regarding their respective solvent requirements. Tumors were measured every 2–3 days with a digital caliper and the volumes were calculated using the eq. V = d_1_ x (d_2_)^2^ /2, (V = volume, d_1_ = larger diameter, d_2_ = smaller diameter). Mice were euthanized using a carbon-dioxide chambers when tumors reached a diameter of 2 cm or became ulcerated. In order to probe for potential drug toxicities, 12 additional naive mice were treated with same treatment regimens described above (*n* = 3/group), and sent for autopsy at the end of the 21-day treatment interval (Division of Comparative Medicine, Washington University). Blood was collected for complete blood count (CBC) and biochemical analysis (AST, ALT, BUN, total bilirubin, and Cr). Organs were examined grossly and histologically.

### PDX model and in vivo assessment of tumor growth and survival

Omental metastatic tumor was harvested from a patient undergoing cytoreductive surgery for ovarian cancer and placed in RPMI on ice. The harvested tumor was divided into four 5 mm tumors and implanted into the right flank of two NSG mice under general anesthesia. Implantation was performed within 20 min of tissue harvest. Once the tumors grew larger than 15 mm, they were harvested and implanted into subsequent NSG mice to generate stable in vivo PDX lines (three passages). Hematoxylin and eosin staining (H&E) of an established PDX tumor was harvested and confirmed its initial characteristics determined at biopsy - high-grade serous adenocarcinoma (Suppl. Fig. S1). This confirmed tumor was then transplanted into the flanks of 25 NOD.CB17-PRKDSCID mice. Tumors were established and treatment started at ~ 150 mm^3^ tumor volume. Mice were randomized into four treatment groups with five mice per group (*n* = 5). The mice then received daily intraperitoneal injections with 100 μL of vehicle (25% cremophor in H_2_O), weekly cisplatin 4 mg/kg, daily SW IV-134 (750 nmoles [26 mg/kg]), and a combination of daily SW IV-134 (750 nmoles [26 mg/kg]) and weekly cisplatin 4 mg/kg for 14 days. Tumors were measured every 3–4 days with a digital caliper and mice were euthanized when tumors reached a cross-sectional diameter of 2 cm or ulcerated**.**

### Statistics

Statistical analyses and data plotting were performed using GraphPad Prism software version 8 (San Diego, CA) and IBM SPSS Statistics 25 (Armonk, NY). Results were expressed as mean ± SEM of at least 3 biological replicates for in vitro data. One-way ANOVA was used to analyze the differences in viability and caspase activity assays. Unpaired two tailed t-tests were used to evaluate the difference in CBC, biochemistry analyses, and to confirm the difference in subgroups. Mixed model two-way ANOVA was used to analyze the difference in tumor sizes in order to adjust for missing data when mice died or were euthanized. Kaplan-Meier survival analysis was used and the difference between the groups was compared with a log-rank test. A *p-*value of < 0.05 was considered significant for all analyses.

## Results

### The targeted SMAC mimetic SW IV-134 is a potent enhancer of cisplatin-induced cell death

**Fig. 1:**
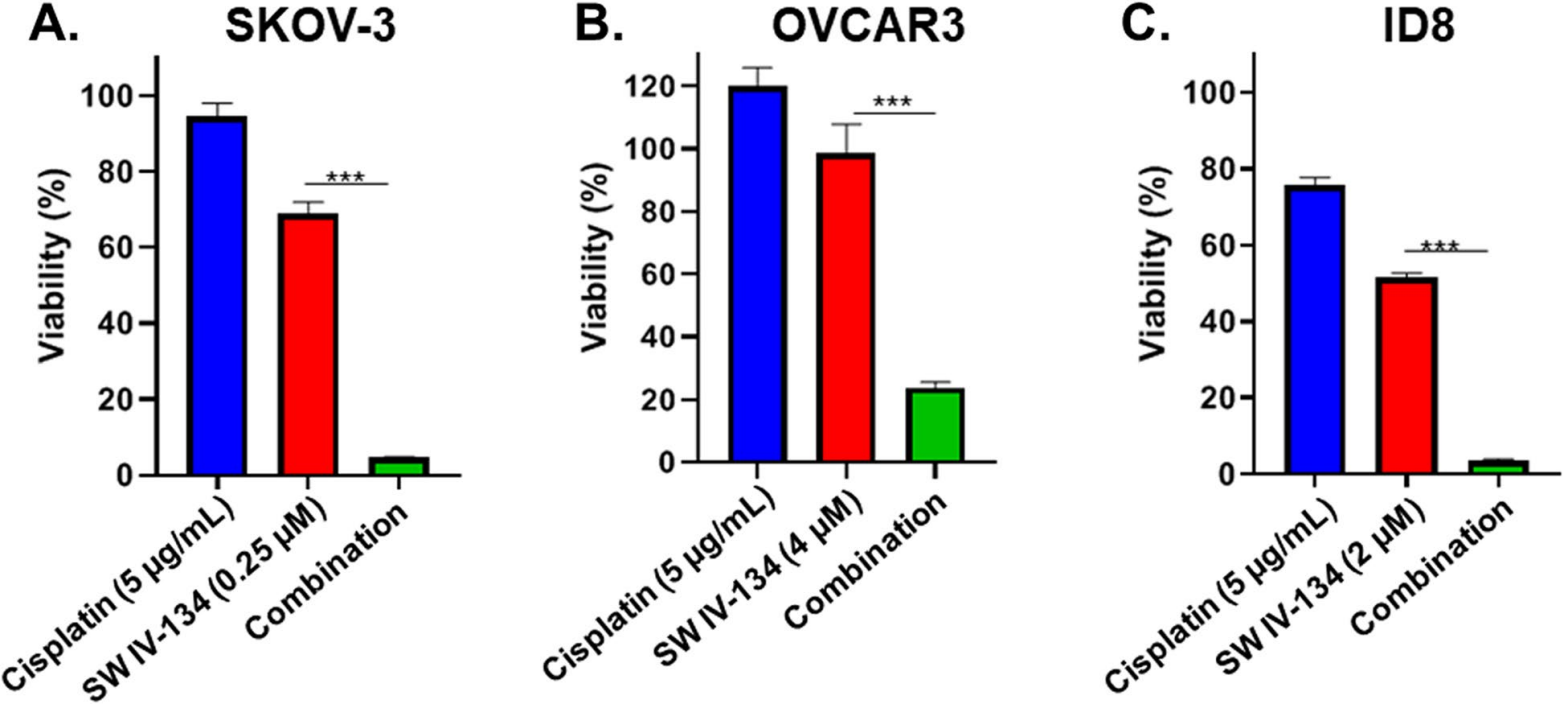
The combination of cisplatin and SW IV-134 shows enhanced reduction in ovarian cancer cell viability. **A**, SKOV-3, **B**, OVCAR-3 and **C**, ID8 cells were treated with cisplatin (5μg/ml), SW IV-134 (varying concentrations), or the combination of the two drugs using the same concentrations. Titer-Glo viability assays were performed after 72 h (SKOV-3 and OVCAR-3) or 36 h (ID8) of treatment. The data were normalized to DMSO treated control cells. (***p < 0.001) (*n* = 3)

Three frequently utilized ovarian cancer cell lines were chosen for our initial treatment assessments. In order to investigate the combined effects of our study drugs, we determined the minimally effective dose of each drug alone in a series of pilot experiments. The drug concentration required to induce limited cell death (50% or less) varied between cell lines and ranged from 0.25 μM (SKOV-3, human) to 4 μM (OVCAR3, human), with ID8 cells (mouse) requiring an intermediate dose of 2 μM (Fig. 1, blue and red bars). To test whether a combination of these sublethal doses would increase cell death beyond single-agent potency, we treated SKOV-3, OVCAR3 and ID8 cells with a combination of both compounds. Indeed, the drug combinations substantially increased the overall cytotoxicity in all cell lines with OVCAR3 cells (20% viability), being less sensitive than SKOV-3 and ID8 cells (5% viability) (Fig. 1A - C, *p* < 0.001 for all analyses). The response to combination treatment was far more pronounced than anticipated, given the modest cytotoxicity of the individual components suggesting a synergistic rather than an additive mode of action.

**Fig. 2:**
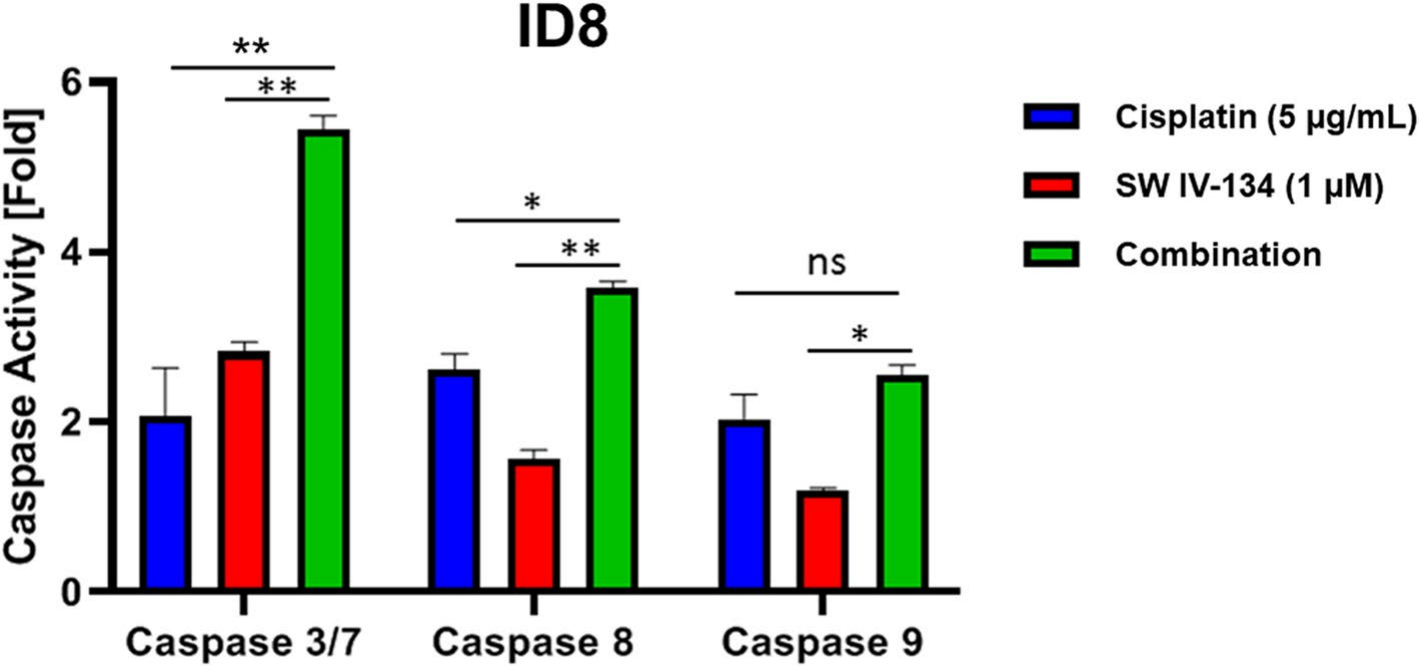
The combination of cisplatin and SW IV-134 leads to augmented apoptotic cell death. Mouse ID8 cells were treated with cisplatin (5 μg/mL), SW IV-134 (1 μM), and a combination of the two drugs at their respective concentrations. The activation status of caspases 3, 8 and 9 were measured using a Caspase-Glo Assay System. The data are normalized to the luminescence signals for each caspase on cells treated with DMSO (baseline) (*n* = 3, * *p* < 0.001, **p < 0.0001, ns = non-significant)

Even though SW IV-134 triggers more complex aspects of the apoptosis machinery, including cIAP degradation, NIK activation and TNF-a production (see Discussion and Refs. [31, 33]), the following experiments were designed to focus on its ability to interfere with XIAP, in effect increasing the activity of intracellular caspases. We therefore studied the relative contribution of drug treatment on the activation of caspases-3/7 (terminal pathway), caspase-8 (extrinsic pathway) and caspase-9 (intrinsic pathway). Using a fluorescence-based caspase activation assay, treatment of ID8 cells with cisplatin and SW IV-134 alone induced only a slight activation process for all caspases ranging from 1.2–2.8-fold over baseline (Fig. 2). Combination of cisplatin and SW IV-134 led to an even further increase in caspase activity (2.5–5.4-fold) and reached the highest levels of activation across all single-agent regimens with one exception - caspase-9/cisplatin (Fig. 2). These data suggest that the strongest impact on overall cell death induction is likely mediated via the terminal apoptosis pathway (executioner caspase-3).

### SW IV-134/cisplatin combination therapy leads to an improved treatment response in an immunocompetent mouse model of ovarian cancer (syngeneic model)

**Fig. 3:**
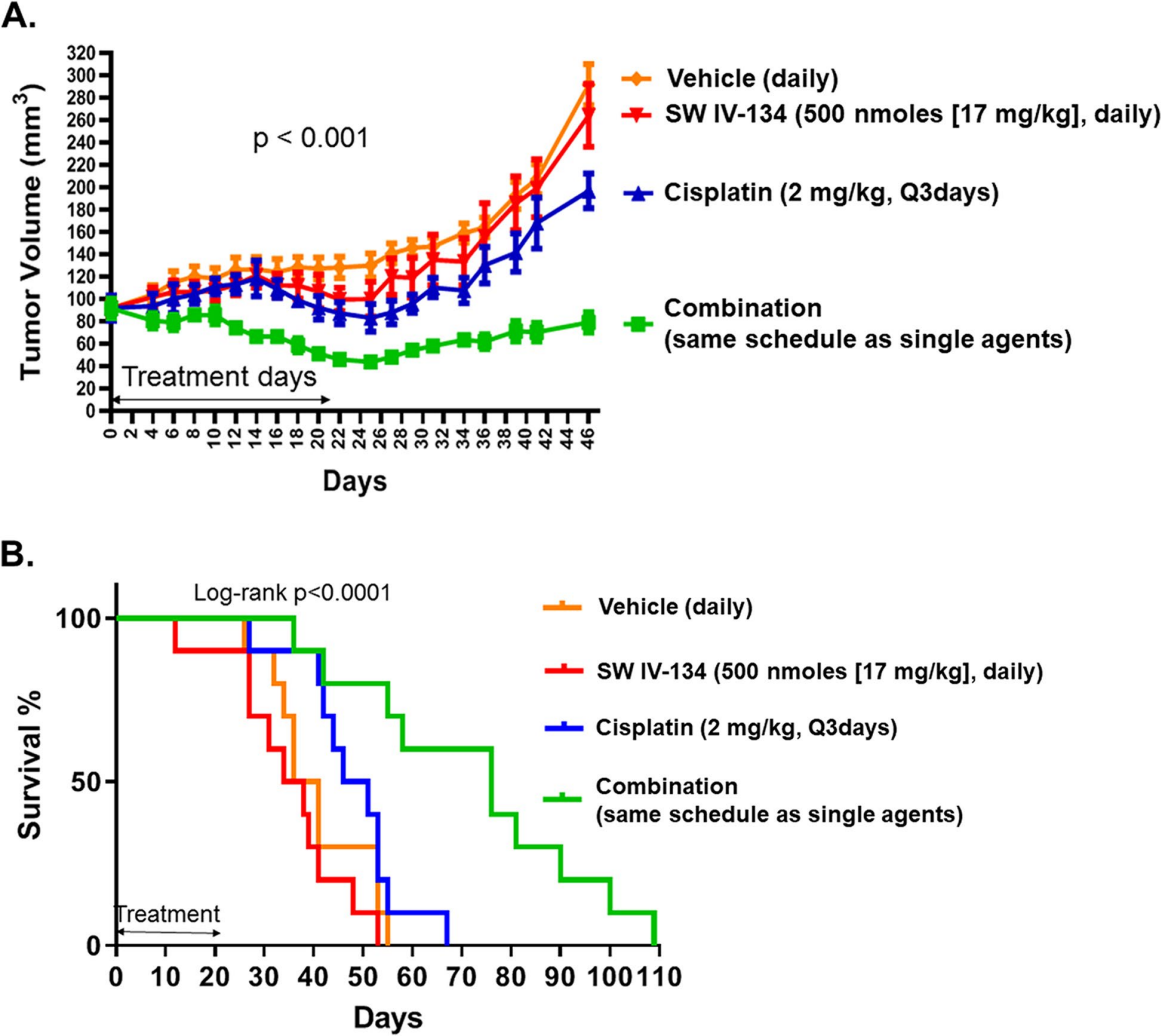
The combination of SW IV-134 and Cisplatin therapy leads to improved objective response rate and survival in an immune-competent ovarian cancer mouse model. An immune-competent allograft mouse model of ovarian cancer was established after right flank injection a 200 μL single cell suspension of 1 × 10^7^ ID8-Luey cells. The mice were treated with the above 4 treatment regimen with vehicle being the control group. **A**, The tumors were measured every other day using digital calipers. The change in tumor volumes between the groups was statistically significant with the tumor volumes of the combination group being significantly lower than vehicle (*p* < 0.0001), SWIV-134 (*p* = 0.01) and cisplatin (*p* = 0.001) at 36 days. **B**, Kaplan-Meier survival curve of mice in (**A**). Survival of the combination treatment group was significantly longer than any other treatment group with median survival being 36, 34, 46 and 76 days in the vehicle, SW IV-134 alone, cisplatin alone and combination treatment groups, respectively (*p* < 0.001)

In order to determine if the drug combination concept observed in vitro would translate to a similar response in vivo, we applied a syngeneic animal model by injecting luciferase-labeled ID8 ovarian cancer cells (ID8-Luey) into the flanks of immunocompetent C57BL/6 mice. The mice were randomized into four groups and a three-week treatment regimen started when tumor volumes reached ~ 100 mm^3^. Mice treated with vehicle served as a control. Both single-agent treatment arms showed little signs of treatment response, reflected by tumor growth patterns similar to the vehicle control. In contrast, the combination group demonstrated a strong treatment response, associated with tumor shrinkage, which started shortly after drug administration (Fig. 3A). About 14 days into the treatment period, both single-agent groups appeared to develop mild treatment responses and a reduction in tumor size. Several days post-treatment cessation, the tumors of all groups started growing again, albeit at differential kinetics, with the control and single-agent groups resuming at a higher growth pace than the combination group (Fig. 3A, *p <* 0.0001). The median survival of the combination group was nearly twice as along (76 days) as the most effective monotherapy (cisplatin, 46 days), followed by vehicle (36 days) and SW IV-134 (34 days), respectively (Fig. 3B, *p* < 0.0001). Of note, two out of ten mice (20%) in the combination group survived for more than 100 days, while no such long-term survivors were identified in any other treatment group.

We did not observe significant differences in complete blood counts or serum chemistry between the treatment groups, indicative of only mild, if any systemic toxicities of drug therapies (Suppl. Table S1). Some mice demonstrated mild irritation or ulcers at the site of peritoneal drug injection as well as slight initial weight loss (SW IV-134). However, this trend did not continue and all mice recovered from this drug effect by day 10 of therapy. In addition, organ analysis (brain, heart, lungs, alimentary tract, kidneys, liver and pancreas) did not reveal signs of adverse drug effects and the absence of discernible change in mouse behavior (failure to groom) and treatment-related deaths further support the notion that SW IV-134/cisplatin combination therapy was well tolerated.

### SW IV-134/cisplatin combination therapy leads to complete tumor eradication in a patient-derived xenograft (PDX) model of ovarian cancer

With the goal of performing a clinically more relevant efficacy model, we successfully generated a patient-derived tumor line in immunocompromised mice using omental tumor tissue obtained from a woman with a fallopian tube carcinoma undergoing cytoreductive surgery. In order for it to be considered a stable PDX line, the initial tumor implant was passaged four times using naïve founder mice. At this point, the tumor was harvested and H&E staining confirmed a high-grade serous carcinoma (Suppl. Fig. S1). Tumor tissues (5 mm) were transplanted into NOD.CB17-PRKDSCID experimental mice. When the tumor volumes reached ~ 150 mm^3^, the mice were randomized and treated using the same conditions and shorter schedule than described above for the syngeneic mouse model.

**Fig. 4:**
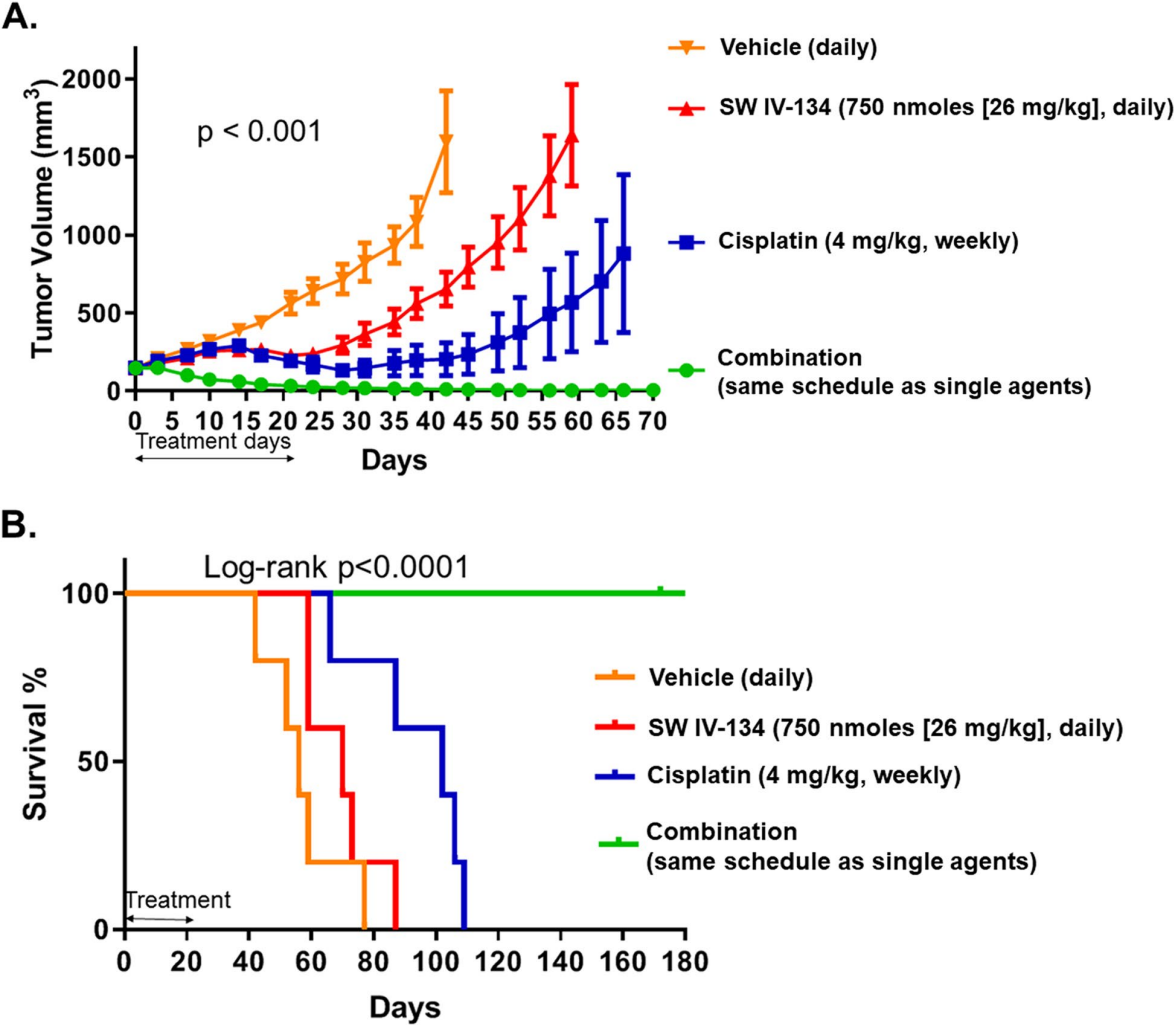
The combination of SW IV-134 and Cisplatin therapy leads to improved complete tumor response rate and survival in a patient-derived xenograft (PDX) model of ovarian cancer. A patient-derived xenograft model of ovarian cancer was established by transplanting 5 × 5 mm tumors into the right flank of immunocompromised NOD.CB17-PRKDSCID female mice. Once growing tumors were confirmed, the mice were treated with the above 4 treatment regimen with vehicle being the control group. **A**, The tumors were measured every other day using digital calipers. The change in tumor volumes between the groups was statistically significant and only the combination therapy group saw a significant reduction in tumor volume as well as 3 complete responses. **B**, Kaplan-Meier survival curve of mice in (**A**). Three mice in the combination therapy group had a complete response and long-term survival until natural cause of death. The median survivals were 56, 70, 102 and 200 days in the vehicle, SW IV-134 alone, cisplatin alone and combination treatment groups, respectively (*p* < 0.001)

Most noticeably, combination therapy showed an immediate and robust response to the drugs and led to a complete disappearance of visible tumors in three of the mice (60%) without signs of disease recurrence throughout their lifetime (Fig. 4A, *p <* 0.0001). Similar to the syngeneic tumor model described above, we noticed some response to the single-agent groups after ~ 15 days of treatment. Shortly after treatment cessation, tumors started growing again with cisplatin alone being somewhat more effective than SW IV-134 alone, illustrated by a more rapid tumor growth curve in the latter group. Three of the mice in the combination group died of natural causes while the median survival of mice treated with vehicle, SW IV-134 alone and cisplatin alone was 56, 70 and 102 days, respectively (Fig. 4B, *p* < 0.0001). We observed some weight loss in the mice treated with Cisplatin but failed to detect abnormalities in mouse behavior (failure to groom) and drug-related deaths throughout the course of the experiment.

## Discussion

In our current study, we have evaluated a novel drug treatment and combination strategy for ovarian cancer. We sought to investigate if cisplatin, an established standard-of-care treatment for Mullerian carcinomas, could be safely and effectively combined with a cancer-targeted SMAC mimetic (SW IV-134) as a means to substantially improve cancer outcomes and toxicities. When used in combination, sublethal doses of cisplatin and SW IV-134 led to substantially increased death pathway activation in vitro, much more so than the individual cancer drugs were able to accomplish in isolation, suggestive of a more than additive effect. Similarly, when tested in vivo employing syngeneic (immunocompetent hosts) and patient-derived xenograft (PDX) models of ovarian cancer (immunocompromised hosts), combination therapy consistently resulted in robust tumor responses and corresponded with greatly improved animal survival when compared to monotherapy control arms. Most noticeably, combination therapy led to complete responses in the PDX ovarian cancer model, in which 60% of the mice were tumor-free and showed no evidence of recurrent disease over the course of their natural lifetime. These pre-clinical studies demonstrate that the combination of cisplatin and SW IV-134 represents a viable and promising treatment strategy for Mullerian carcinomas, which include ovarian, fallopian and primary peritoneal carcinomas.

Platinum-based medications have been safely combined with other chemotherapeutics in the primary treatment of Mullerian carcinomas [5, 34–36]. In cases where the cancers recurs less than 6 months from completion of chemotherapy, platinum-based chemotherapy is usually discontinued, unless evidence of resistance reversal is presented [37]. Since subsequent treatment regimens are usually associated with minimal efficacy and increased toxicities, we are in dire need of innovative and novel treatment strategies for recurrent Mullerian carcinomas [34–36]. Our research has demonstrated that low-dose SW IV-134/cisplatin combination therapy resulted in better treatment outcomes than merely the sum of its individual components, indicative of a synergistic drug interaction in the absence of overt toxicities.

With respect to ovarian cancer in particular, overexpression of inhibitor of apoptosis proteins (IAPs) contribute to a significant degree of drug resistance by preventing efficient activation of apoptotic cell death [17–19, 38]. XIAP and cIAP are the most prominent and potent members of this family and its pharmacologic blockade with SMAC mimetics has been shown in a number of experimental settings [39, 40] but also as a means to sensitize ovarian cancer efficiently to chemotherapy [25–29, 41], including in a clinical setting [42]. We have previously shown that the conjugate SW IV-134 leads to rapid cell death via activation of caspases, degradation of cIAP-1, cIAP-2, activation of NF-қβ and induction of TNF-α [31, 33]. As a result, our prior research has indicated that this drug conjugate exerted increased activity against ovarian cancer in vitro and in vivo, and sensitized chemo-resistant pancreatic cancer to gemcitabine-based combination therapy [30–33, 43]. Our next steps would be to study the role of SW IV-134 in sensitizing chemotherapy resistant ovarian cancer to platinum-based chemotherapy, since resistance to platinum-based chemotherapy is one of the most important prognostic factors for this disease.

Therefore, restoring the ability to undergo programmed cell death by inhibiting XIAP and activating TNF-α via cIAP degradation appears to be an attractive strategy for the treatment of Mullerian carcinomas. In order to most effectively target ovarian cancer cells and decrease systemic toxicities, the delivery of the XIAP antagonist has been rendered cancer selective by linking the SMAC mimetic to the sigma-2 ligand SW43, the receptors of which are upregulated in ovarian cancer cells [30]. This treatment concept uses targeted therapeutics capable of delivering the cytotoxic agents directly into the cancer cells [32] and requires less drug to accomplish the same biologic effects the non-targeted compounds can only achieve at a much higher dose. Here, we have also shown that this novel drug can be safely used in combination with standard of care platinum-based chemotherapy with a trend toward synergistic tumor eradication and limited overall systemic toxicities.

## Conclusions

Future studies are highly warranted to test our particular drug combination to obtain evidence for overcoming apoptosis-related platinum resistance in Mullerian carcinomas using additional chemotherapy resistant ovarian cancer but also fallopian or primary peritoneal cancer cell lines as well as patient-derived tumors. Platinum-resistant and refractory ovarian cancer has a very poor prognosis with an overall survival of months, and novel therapeutic approaches in this arena are thus desperately needed. Given that combination therapy significantly decreased the tumor burden in immunocompetent as well as in the clinically relevant patient-derived xenograft models of ovarian cancer, resulting in complete treatment responses, we propose that this drug combination should be tested more broadly in PDX-based animal models before advancing toward clinical trials.

## Supplementary Information

**Additional file 1.** Supplementary information.

## Data Availability

All data reported in this manuscript are available from the corresponding author upon reasonable request.
