# Supplementary material for: The targeted SMAC mimetic SW IV-134 augments platinum-based chemotherapy in pre-clinical models of ovarian cancer
Source: BMC Cancer. 2022 Mar 12;22:263. doi: 10.1186/s12885-022-09367-w (PMC8918278; doi:10.1186/s12885-022-09367-w)
Supplement: Supplementary file 1 — Additional file 1. Supplementary information. [file 12885_2022_9367_MOESM1_ESM.pdf]

# The targeted SMAC mimetic SW IV-134 augments platinum-based chemotherapy in pre-clinical models of ovarian cancer

Pratibha S. Binder[1†], Yassar M. Hashim[2††], James Cripe[1], Tommy Buchanan[1], Abigail Zamorano[1], Suwanna Vangveravong[2], David G. Mutch[1,3], William G. Hawkins[2,3], Matthew A. Powell[1,3] and Dirk Spitzer[2,3*]

## SUPPLEMENTARY INFORMATION

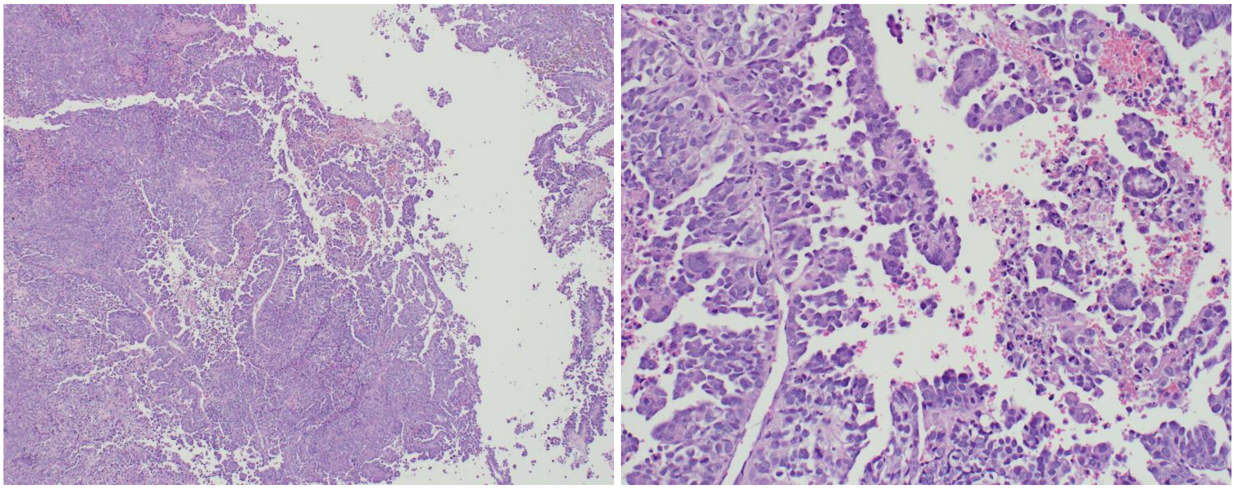

**Figure S1. Hematoxylin and eosin staining of paraffin embedded tissue block of tumor obtained from passage #4 of the patient-derived xenograft.** The block shows a high-grade serous adenocarcinoma at 40X (left) and 200X (right) magnification.

| Mouse Treatment | WBC ($10^3$ /µL) | HGB (g/dl) | Platelets (103 /µL) | AST (u/L) | ALT (u/L) | BUN (mg/dL) | Creatinine (mg/dL) |
|---|---|---|---|---|---|---|---|
| Combination | 7.58 | 11.8 | 1112 | 78 | 46 | 20 | 0.34 |
| Combination | 4.76 | 12.3 | 916 | 94 | 62 | 25 | 0.32 |
| Combination | 5.54 | 12.7 | 849 | 69 | 64 | 21 | 0.31 |
| Cisplatin | 8.74 | 13.0 | 846 | 78 | 43 | 26 | 0.28 |
| Cisplatin | 4.64 | 12.9 | 818 | 44 | 40 | 29 | 0.31 |
| Cisplatin | 2.62 | 12.5 | 483 | 106 | 56 | 32 | 0.38 |
| SWIV-134 | 7.08 | 13.1 | 955 | 271 | 54 | 29 | 0.32 |
| SWIV-134 | 4.66 | 12.0 | 1021 | 123 | 37 | 23 | 0.27 |
| SWIV-134 | 4.56 | 12.7 | 966 | 52 | 41 | 30 | 0.32 |
| Control | 7.4 | 13.4 | 930 | 86 | 41 | 31 | 0.35 |
| Control | 6.42 | 12.9 | 888 | 44 | 42 | 32 | 0.33 |
| Control | 8.08 | 13.4 | 904 | 105 | 46 | 29 | 0.33 |

**Table S1. Combined cisplatin and SW IV-134 does not have any significant effect on complete blood count or serum chemistry in immune-competent mice.** Complete blood count (CBC) and serum chemistry analysis of 3 immunocompetent mice per treatment group was performed after 21 days of treatment. There is no statistically significant difference in the laboratory values between the groups. (WBC, white blood cell count; HGB, hemoglobin; AST, aspartate aminotransferase; ALT, alanine aminotransferase; BUN, blood urea nitrogen**;** Cr, Creatinine)
